# Unraveling the Association Between Schizophrenia and Substance Use Disorder-Predictors, Mechanisms and Treatment Modifications: A Systematic Review

**DOI:** 10.7759/cureus.16722

**Published:** 2021-07-29

**Authors:** Anum Masroor, Arseni Khorochkov, Jose Prieto, Karan B Singh, Maduka C Nnadozie, Muhammad Abdal, Niki Shrestha, Rose Anne M Abe, Lubna Mohammed

**Affiliations:** 1 Psychiatry, California Institute of Behavioral Neurosciences & Psychology, Fairfield, USA; 2 Psychiatry, Psychiatric Care Associates, Englewood, USA; 3 Medicine, Khyber Medical College, Peshawar, PAK; 4 Internal Medicine, California Institute of Behavioral Neurosciences & Psychology, Fairfield, USA; 5 Research, California Institute of Behavioral Neurosciences & Psychology, Fairfield, USA; 6 Emergency Medicine, California Institute of Behavioral Neurosciences & Psychology, Fairfield, USA

**Keywords:** schizophrenia, substance use disorders, cannabis use, drug addiction, gene-environmental

## Abstract

Individuals with schizophrenia are particularly vulnerable to substance abuse problems. Comorbidity with substance use disorders (SUDs) frequently results in early death and increased dysfunction observed in schizophrenia. This dual diagnosis can be explained through multiple general mechanisms. Tobacco, alcohol, cannabis, and cocaine are substances widely used by individuals with schizophrenia. This study highlights the predictors, mechanisms responsible for the relationship between substance use disorder and schizophrenia and how it can help with the treatment of both disorders. The publications were rigorously reviewed after being found in multiple databases. The study's inclusion criteria were research published within the last five years, publications written in English, full-text availability, and human studies. A total of ten papers were selected for examination from a total of 9,106 articles found using the search method across several databases. This study follows the rules listed within the Preferred Reporting Items for Systematic Reviews and Meta-Analyses (PRISMA) checklist 2009. The information gathered from these published studies was used to investigate the elements that contribute to the link between schizophrenia and substance abuse. Here, we evaluate a close relationship between schizophrenia and substance use disorders. The articles studied exhibit a bidirectional association between the two disorders in most individuals. From our analysis, the comorbidity between the two disorders is partially due to shared polygenic liability. Individuals with schizophrenia have dysfunctional Mesocorticolimbic brain reward circuits indicating a history of substance use. An underlying genetic vulnerability to schizophrenia may be triggered by extensive cannabis usage at a young age. A combination of psychological and pharmacological interventions for both disorders can significantly improve the outcome.

## Introduction and background

Schizophrenia is a serious psychiatric illness affecting 1% of the population around the globe [1]. It is a severe and complex mental health illness marked by a lack of feeling, incapacity, changes in thought, insight, and behavior. Many patients have delusions, hallucinations, and misperceptions of reality [2]. It frequently results in a lifetime of disability and cognitive impairment [3]. Substance use disorders are defined as conditions in which the patient's misuse or addiction to substances such as alcohol, cannabis, cocaine, nicotine, opioids, phencyclidine, or amphetamine, among others, has had a detrimental effect on their family and social lives, work, or school, or has resulted in financial difficulties [2]. Patients with schizophrenia are more likely to develop substance abuse problems [1]. In one epidemiological investigation, 47% of schizophrenia patients were found to have a substance abuse problem [3]. Comorbidity with substance use disorders (SUDs) frequently results in early death and increased dysfunction observed in schizophrenia [4]. This dual diagnosis can be explained through multiple general mechanisms: a. substance use disorder may develop as a result of schizophrenia, b. drug use disorder could be one of the causes of schizophrenia, or c. both diseases could share the same underlying risk factors, such as environmental and genetic variables [5]. Tobacco, alcohol, cannabis, and cocaine are substances widely used by individuals with schizophrenia [6]. Substances regularly taken by schizophrenia patients have been shown to have direct effects on the parts of the brain thought to be affected by disease processes in psychosis [7]. Substance misuse not only appears to increase the risk of acquiring psychotic symptoms, but it also tends to have a negative impact on the course of schizophrenia, with patients reporting more positive symptoms, greater rates of treatment noncompliance, and higher relapse rates [8]. The correlation between schizophrenia and substance use disorder has been investigated to a great extent in multiple population-based studies. There is reliable proof indicating a causal, dose-dependent relationship between substance use disorders and the onset of schizophrenia if the onset of substance use disorders predates the onset of schizophrenia [2]. Many studies have shown that for patients with comorbid diagnoses of a serious psychiatric illness and a substance use problem, treatment of the psychiatric illness improves the outcome of the mental disorder and occasionally that of the substance use disorder as well [6]. Most of the studies either focus on the genetic mechanism or environmental factors responsible. To our knowledge, not many studies are available that include details of all the factors responsible for this comorbidity and how it impacts the treatment course. The purpose of our study is to write a detailed literature review that describes the predictors, mechanisms responsible for determining the link between substance use disorder and schizophrenia and how it can help with the treatment of both disorders.

Methods

We conducted a scientific review following Preferred Reporting Items for Systematic Reviews and Meta-Analyses (PRISMA) guidelines. A search of the database, PubMed, PubMed Central, Medline, Google Scholar, and Science Direct was conducted up to May 6th, 2021. The search for relevant studies using generic keywords (“Schizophrenia” AND Substance use disorder”) was done. The relevant Medical Subject Headings (MeSH) terms and keywords “Schizophrenia,” “substance use disorder, psychology,” “physiopathology” “pathology,” “complications,” and “diagnosis” were used in various combinations using Boolean operators like “AND” and “OR,” and 1,074 relevant studies were identified, with a total of 9,106 studies. Table 1 and Table 2 summarize the search strategy using keywords and MeSH terms, respectively.

**Table 1:** Search strategy using keywords

Keyword	Total articles	2016-2021 (5 years)	Free Full text
Schizophrenia and substance use disorder	8,024	1,224	532

**Table 2:** Search strategy using MeSH terms MeSH: Medical education Subject headings

MeSH strategy		DATABASE		RESULTS	
"Schizophrenia/pathology"[MeSH] AND "Schizophrenia/psychology"[MeSH]		PubMed		469	
"Schizophrenia/complications"[MeSH] AND "Schizophrenia/diagnosis"[MeSH]		PubMed		90	
"Substance-Related Disorders/complications"[MeSH] AND "Substance-Related Disorders/diagnosis"[MeSH]		PubMed		216	
"Substance-Related Disorders/physiopathology"[MeSH] AND "Substance-Related Disorders/psychology"[MeSH]		PubMed		290	
"Schizophrenia/complications"[MeSH] AND "Substance-Related Disorders/diagnosis"[MeSH]	PubMed		4		
"Substance-Related Disorders/complications"[MeSH] AND "Schizophrenia/diagnosis"[MeSH]	PubMed		5		

 

**Figure 1:**
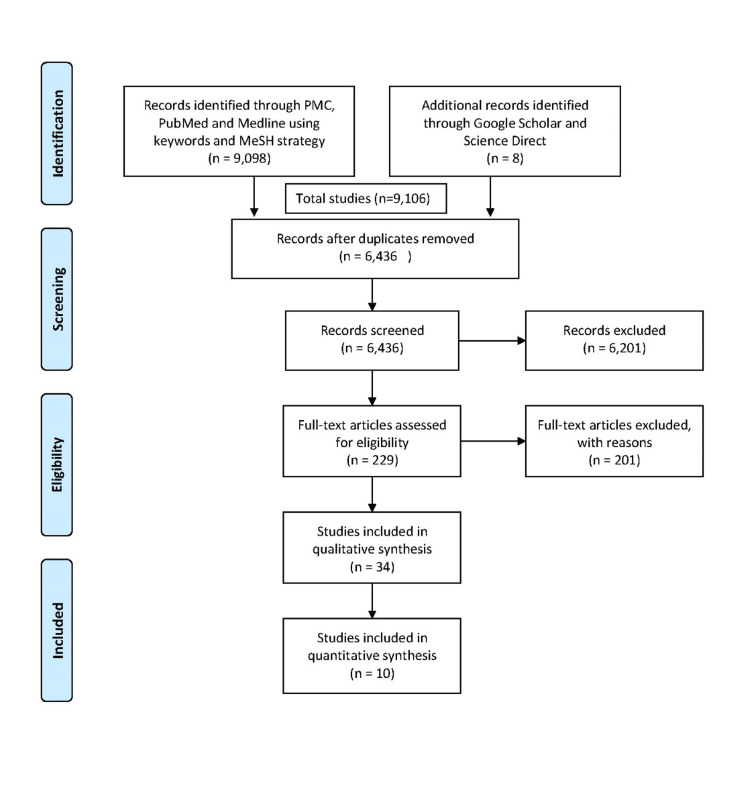
PRISMA flow diagram outlining the search process PRISMA: Preferred Reporting Items for Systematic Review and Meta-Analyses, PMC: PubMed Central

The inclusion/exclusion criteria were applied, and records from January 2016 to May 2021 were identified. The search included the studies published in the English language for human participants. The duplicate articles were removed after inclusion criteria were applied. Gray literature wasn't included during this study per inclusion/exclusion criteria. Preferred Reporting Items for Systematic Reviews and Meta-Analyses (PRISMA) flowchart diagram of literature retrieval is shown in Figure 1.

Results

From the databases, a total of 9,106 studies were discovered. Filters were used to eliminate duplicates and narrow the number of articles to 6,436 based on inclusion criteria (full-text studies in English, published within the previous five years, on humans, scientific trials, all types of reviews, observational studies), and duplicates were removed. The publications were screened based on their titles, and 229 relevant studies were kept. Abstract and full-text articles were reviewed, and relevant studies came down to 34. All research was evaluated for quality, and the number of papers included was reduced to ten. These included four literature reviews, three case-control studies, two cross-sectional studies. Two authors independently checked the quality of the articles using the following quality assessment tools: Newcastle-Ottawa tool and Scale for the Assessment of Narrative Review Articles (SANRA) checklist. The study characteristics are summarized in Table 3.

**Table 3:** Characteristics of the included studies (n=10) SUDs: Substance Use Disorders, SSDs: Schizophrenia Spectrum Disorders

Study	Authors	Year	Type of study	Patients	Purpose of the study	Conclusion
1.	Manseau et al. [6]	2016	review		Etiology of comorbidity and effect of treating both conditions simultaneously	The etiology of dual diagnosis is multifactorial. Treatment of both schizophrenia and SUDs improves functional and clinical outcomes
2.	Crockford et al. [8]	2017	review		To find evidence-based approaches that help people with schizophrenia and substance use disorders live better lives	Best results are obtained when antipsychotic drugs are used in conjunction with addiction-based psychosocial therapies
3.	Polimanti et al. [4]	2017	review		Discuss the results of recent genetic studies on dual diagnosis	Schizophrenia and substance use disorder share a hereditary connection and common genetic variants
4.	Hartz et al. [5]	2017	Case-control study	1,929	To determine the relation between schizophrenia polygenic risk scores and substance dependence	The comorbidity is partially attributable to shared polygenic liability
5.	Cederlöf et al. [9]	2017	Cohort study	9,242	To determine the association between psychosis and development of SUDs later in life and/or suicide attempts	Dose response relationship was found between psychosis and future substance use disorder and/or suicide attempts
6.	Quinn et al. [7]	2018	Case-control study	158	Examine the regional gray matter volume differences between patients with schizophrenia who use substances and those who don't	The gray matter abnormalities related with schizophrenia are not notably revealed by a clinically important history of substance use
7.	Vaucher et al. [10]	2018	Case-control study	34,241	Genome-wide association study to clarify the causal role of cannabis consumption on the risk of schizophrenia	The genetic approach backs the underlying role of cannabis use on the risk of schizophrenia
8.	Khokhar et al. [1]	2018	review		To determine whether the genetic factors that influence risk for schizophrenia make patients vulnerable to substance use	A malfunctioning Mesocorticolimbic brain reward circuit may result from a genetic or early environmental injury, resulting in increased substance use, and substance use may induce the onset of schizophrenia
9.	Tumenta et al. [3]	2020	Cross-sectional	349	To determine the prevalence of substance abuse among patients with schizophrenia spectrum disorders (SSDs) at a community teaching hospital	There is a strong link between substance use and schizophrenia spectrum disorders (SSDs), with 75% of SSD patients using a substance
10.	Patel et al. [11]	2021	Cross- sectional	1,030,949	To see if cannabis use issues lead to medication non-adherence in people with schizophrenia	Cannabis use disorder was found to be a significant risk factor for medication non-adherence in schizophrenia patients

## Review

Substance use disorders are associated with serious adverse consequences, among patients with schizophrenia including worse psychiatric symptoms, decreased functioning, and increased medical illness and death compared with schizophrenia patients without co-existing substance use. The etiology of this association is multifactorial, involving neurobiological, genetic, and environmental factors [6].

Which comes first?

**Figure 2:**
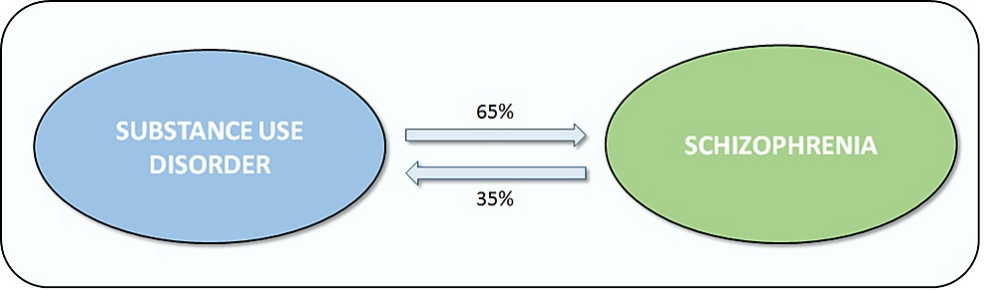
Bidirectional association between substance use disorder and schizophrenia

Patients with psychotic diseases have a greater lifetime risk of substance use disorders [1]. Rates of substance use in patients with psychosis as the predominant symptom range from 30-70 percent [12]. Patients with schizophrenia had a greater rate of tobacco smoking prior to the onset of the illness than those who did not have the condition. Furthermore, several studies have found that consuming cannabis and possibly cigarettes as a teen increases the likelihood of developing schizophrenia [1]. A recent meta-analysis on cannabis verified its potential role: In a dose-dependent way, increased cannabis usage was linked to a higher risk of psychosis, with heavy users face a four-fold danger, while moderate users face a two-fold risk [13]. While cannabis use by teenagers is a significant risk factor for psychosis, other contextual and ecological factors significantly influence the chance of developing schizophrenia [14]. Childhood trauma and cannabis use, for example, appear to work in tandem to increase the risk of psychosis later in life [15]. Psychotic symptoms like hallucinations and delusions in teens are linked with the development of substance use disorder later in life [9]. Not everyone who develops schizophrenia uses cannabis (or other narcotics) prior to the onset of psychotic symptoms. Surprisingly, a meta-analysis indicated that teen alcohol use had no effect on the age at which psychosis began [1]. Another study showed that the majority of people with comorbid drug use disorders (65%) were detected before they developed schizophrenia, implying that substance use disorders may have caused schizophrenia to develop. The emergence of substance use disorders followed the onset of schizophrenia in the remaining individuals (35%). This suggests that there may be a bidirectional relationship between substance use problems and schizophrenia as shown in Figure 2 [2].

Genetic association

Genome-Wide Association Study (GWAS) of substance abuse revealed evidence of local hereditary intersection and identified risk genes previously associated with schizophrenia risk [16]. The results of this study were utilized to run polygenic risk score (PRS) analysis to look into the universal chromosomal overlap between schizophrenia. For instance, data from a study of cannabis use suggests the existence of shared genetic processes in these two traits [17,18]. The relationship between schizophrenia PRSs and numerous SUD-related features was investigated in a PRS study conducted by deCODE Genetics; the study shows extensive relation for disorders of alcohol, stimulants, hallucinogens, premature commencement of drug use, sedative use, smoking initiation, and admissions to a hospital for in-patient dependence treatment [19]. Collectively, these outcomes specify that the simultaneous diagnosis of schizophrenia and SUDs is linked to shared covariance resulting from common genomic difference. However, no judgments can be made about the mechanisms that link these complex qualities based on these genetic relationships alone [4]. Mendelian Randomization (MR) methods were utilized in two studies to investigate causation in the relationship between cannabis use and schizophrenia [10,20].

When tobacco smoking and pleiotropy were taken into consideration, Vaucher et al. found that the top ten loci from a GWAS of cannabis usage were connected to a bigger risk of schizophrenia [10]. The study by Gage et al. reported that the risk of schizophrenia is mildly due to genetic predisposition to cannabis usage [20]. Carey et al. study show that predisposition to comorbid schizophrenia and substance use disorder may be influenced by hereditary factors. Indeed, cannabis, cocaine, nicotine, and excessive alcohol use are all connected to polygenic risk scores for schizophrenia [21]. Several genetic variants of the brain-derived neurotrophic factor (BDNF) protein are linked to comorbid schizophrenia and alcoholism, but not to alcoholism alone, proposing that these genetic variations may predispose to these comorbid disorders [22]. Hartz et al. established that there is a strong relationship between the schizophrenia polygenic risk score and substance use disorder and when the statistics were examined to compare any substance use disorder versus no substance use disorder, a solid connection with the schizophrenia polygenic risk scores was seen [5]. However, the results of the Kerner et al. study show that when compared to random cases in the mixed sample, a family history of schizophrenia was not related with an elevated risk of substance use. According to this study, the genetic risk factors for schizophrenia and substance addiction issues are not always linked [2].

Changes in brain volume and circuits

**Figure 3:**
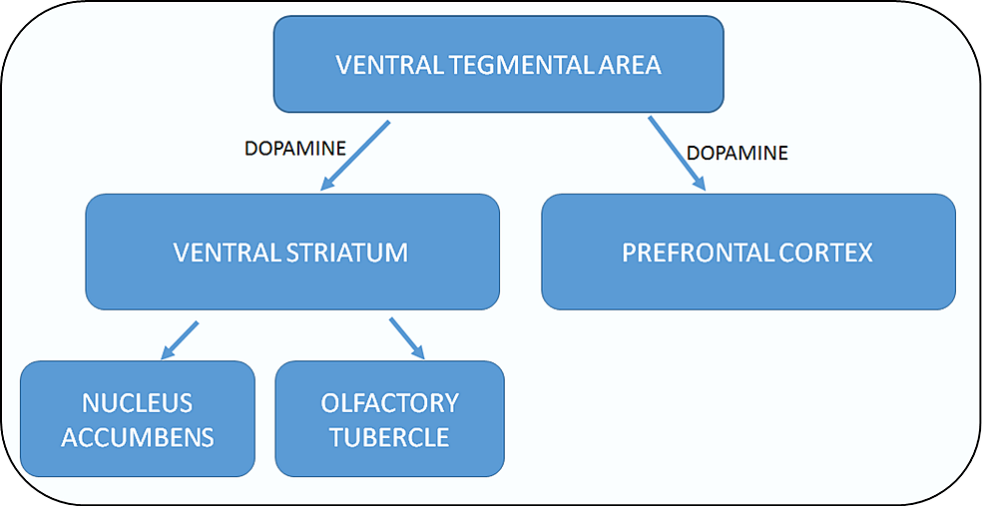
Mesocorticolimbic dopamine circuit

Some neuroimaging studies have suggested that substance abuse complicates research into the neurological underpinnings of schizophrenia [7,23]. Quinn et al. study observed gray matter volume deficits in all expected regions, typically observed in Voxel-Based Morphometry (VBM) studies of schizophrenia except in the cingulate and insula. However, the significant differences in gray matter volume of patients with schizophrenia were not necessarily related to cannabis and alcohol use disorders in the past. Even when patients had solely abused cannabis or alcohol, the results were the same. The use of a region of interest analysis to detect substance-induced volumetric alterations in brain regions affected by psychosis may be more sensitive [7]. This may be most useful for cannabis users with high levels of cannabinoid receptors [24]. The study specifies that the gray matter abnormalities linked with schizophrenia are not significantly shown in clinically relevant histories of alcohol or cannabis use [7]. One symptom of genetic susceptibility to schizophrenia could be disruption in reward and motivation brain circuits (particularly the Mesocorticolimbic dopamine circuitry), which could drive both the onset and continuation of substance use. Dopaminergic activity in the ventral striatum is linked to reward processing in healthy people [1]. Figure 3 shows the Mesocorticolimbic dopamine pathway in healthy individuals [25].

**Figure 4:**
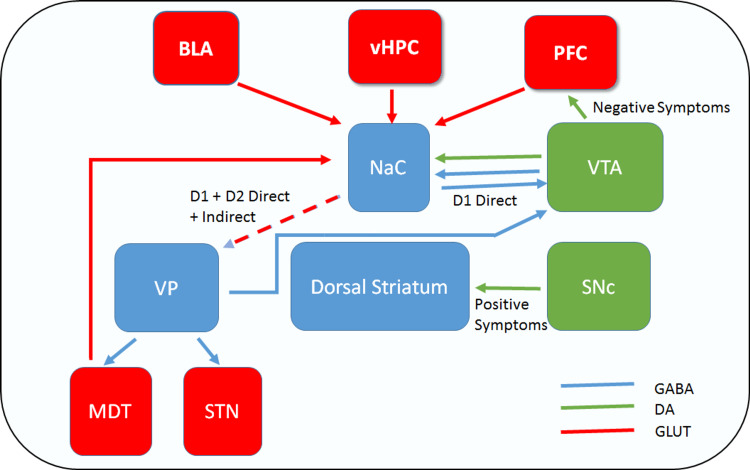
Schematic of brain reward circuitry and dysfunction in schizophrenia and addiction Dopaminergic (Green) and glutamatergic (Red) inputs converge on γ-aminobutyric acid (GABA) ergic (Blue)  neurons in the nucleus accumbens (NAc). These inputs coordinate and regulate direct and indirect outputs that contribute to reward-related behaviors. In schizophrenia, dopamine (DA) signaling is reduced in the cortex, resulting in negative symptoms. However, DA is increased in the striatum, particularly in the dorsal striatum, contributing to the positive symptoms of schizophrenia. BLA = basolateral amygdala; D1 = dopamine type 1 receptor; D2 = dopamine type 2 receptor; PFC = prefrontal cortex; vHPC = ventral hippocampus; VP = ventral pallidum; VTA = ventral tegmental area, MDT = mediodorsal thalamus, STN = subthalamic nucleus, SNc = Substantia Nigra Pars Compacta.

Striatal dopamine release is decreased in patients with schizophrenia and a co-occurring substance use disorder, according to the study, which could be attributable to the patients' substance use histories [1]. Multiple internal circuits within the reward circuit are capable of positive and negative feedback. Human imaging studies, pharmacology, and preclinical trials have shown dysfunction in the striatum, cortex, and hippocampus, involving dopaminergic and glutamatergic signaling respectively in individuals with schizophrenia [26]. According to recent genetic studies, functional alterations in the Mesocorticolimbic dopamine pathway have been linked to several facets of addiction, including relapse and craving [27]. Figure 4 shows the brain reward circuit in schizophrenia and addiction [26,27].

 

Environmental factors

Polimanti et al. study shows that frequent cannabis use at a young age may be linked to a hereditary susceptibility to schizophrenia. Increased genetic predisposition to schizophrenia was linked to a decrease in cortical thickness in males, which could be an endophenotype (i.e., a characteristic that should be heritable, should co-segregate with a psychiatric problem but be present even when the sickness isn't and will be detected at a higher incidence in non-affected family members than in the general population), but only as a result of higher cannabis usage throughout adolescence [4]. Concerning the use of various substances, Olayinka et al. found that patients with Schizophrenia Spectrum disorder (SSD) who had positive cannabis exposure had an increased chance of using other substances paralleled with non-cannabis exposed patients with SSD. The adjusted odds of cocaine, tobacco, and alcohol use were 1.5 to 3 times higher among those with cannabis use compared with those without it [28]. Tumenta et al. study indicates that gender is a particularly important factor, for both substance abuse and schizophrenia, and the use of each class of drugs was more in males as compared to females. The most commonly abused drug, however, was cannabis (28%), followed by alcohol (21%), with fewer individuals using cocaine, hallucinogens, and stimulants [3]. Additionally, in 60-90% of patients with schizophrenia and other psychotic disorders, cigarette smoking has been recognized [8]. Hunt et al. conducted research analysis and found that SUDs are extremely predominant in schizophrenia. The study reported that the prevalence of any SUD was 41.7%, followed by illegal drugs with 24.3%, stimulant use, 26.2%, alcohol and 27.5%, cannabis with 7.3% of the study population [29].

Treatment modifications

Integrated treatment models were established in the 1980s and 1990s and continue to be expanded today. In integrated dual-diagnosis treatment (IDDT), psychiatric and substance use disorders are treated in a single program by a group of clinicians with knowledge of both disorders. IDDT models have typically stressed outreach into the public through services such as case management. Substance use disorder treatments include both pharmacological and psychosocial treatments, which typically use well-known, evidence-based dependence treatment models but may be changed for use with co-occurring disorders [6].

Bahorik et al. study indicates that motivation is a strong predictor of recovery and outcome in schizophrenia, substance-abusing schizophrenia patients have lower motivation levels, implying that they are more likely to have poor outcomes and have more difficulty achieving and maintaining treatment goals than those without the disorder. Furthermore, substance-abusing schizophrenia patients are less motivated to improve their substance-related behaviors and are more likely to relapse than non-schizophrenic substance users [30]. Dual diagnosis patients are less interested in changing their consumption pattern, are more difficult to treat, make slower progress, are more likely to abandon long-term treatment, and are still in danger of return even after years of full remission [31]. Cannabis use disorder significantly increases the likelihood of medication non-adherence in people with schizophrenia [11].

Another major issue is the impact of antipsychotic medicines on these people's substance use. With the exception of clozapine, which has been shown in several studies to reduce substance use in schizophrenia patients across a wide spectrum of substances like alcohol and cannabis, most antipsychotics do not reduce substance use in schizophrenia patients. When clozapine and risperidone were compared in individuals with cannabis use disorder and schizophrenia, it was discovered that because of its ability to adjust attentional bias to drug signals and its activities on lowering activation within the brain reward circuit, clozapine was more successful at reducing cannabis usage [1,32]. 

Kowalczyk et al. study shows that many smokers with schizophrenia have a poor comprehension of the detrimental impacts of smoking, with just 40% of that group fully comprehending the potential for health problems. More research is needed on scholarly programs and individualized psychosocial treatments based on how well different clinical subgroups grasp the dangers of smoking [23].

Although individuals with schizophrenia and concurrent substance use disorders frequently seek treatment in professional settings, there is a paucity of research on the utilization of specific pharmacotherapies or psychosocial interventions for people with dual diagnoses. Best practices include evaluating substance use patterns and integrating care to fit treatment needs to the severity of both disorders and stage of transformation. Although treating persons with schizophrenia with concurrent substance use disorders can be difficult, the statistics show that treatment is effective, and there is a lot of hope for even better results after substance use is ceased [8].

Limitations

Our research has some limitations. To begin, this systematic review includes mixed studies with variability in sample size, control groups, follow-up period, and randomization, which could lead to reporting bias in the real connection between schizophrenia and substance use disorder. Second, we selected studies from 2016 to 2021, this could lead to the omission of crucial data from prior investigations. Third, we were unable to ascertain whether substance use problems predicted the course of schizophrenia or its prognosis, or vice versa. Also, most studies included in this article were review articles based mostly on multiple hypotheses, therefore more clinical trials are needed for stronger evidence.

## Conclusions

Evidence supports a shared vulnerability between patients with substance use and schizophrenia. Bidirectional association between the two disorders has been witnessed in most individuals. With respect to gender, males used more substances than females. The most commonly abused drug was cannabis followed closely by alcohol. Shared polygenic liability is somewhat responsible for the comorbid diagnosis of schizophrenia and substance use disorder. There is a strong relationship between the schizophrenia polygenic risk score and substance use disorders like cannabis use, severe alcohol use and cocaine use. Patients with schizophrenia have dysfunctional Mesocorticolimbic brain reward circuits implying that these people have a history of substance abuse. An underlying genetic vulnerability to schizophrenia may be triggered by extensive cannabis usage at a young age.

However, the gray matter abnormalities associated with schizophrenia are not considerably exacerbated by a history of substance abuse. Also, a family background of schizophrenia was not associated with an increased risk of substance use. Treatment data when it comes to the utilization of specific pharmacotherapies or psychosocial interventions for patients with dual diagnosis is limited, greater improvement is seen when substance use is stopped. Best treatment methods include evaluation of substance use patterns, and synchronized care, addressing the severity of both disorders. We couldn't figure out whether substance addiction disorders predicted the severity and outcome of schizophrenia or the other way around. Future research could shed insight on the underlying molecular mechanisms of these comorbid diseases, as well as provide a variety of treatment alternatives for these difficult-to-treat co-existing illnesses.
